# Titania-Containing Bone Cement Shows Excellent Osteoconductivity in A Synovial Fluid Environment and Bone-Bonding Strength in Osteoporosis

**DOI:** 10.3390/ma14051110

**Published:** 2021-02-27

**Authors:** Tomotoshi Kawata, Koji Goto, Masashi Imamura, Yaichiro Okuzu, Toshiyuki Kawai, Yutaka Kuroda, Shuichi Matsuda

**Affiliations:** 1 Department of Orthopedic Surgery, Kyoto University, Kyoto 606-8507, Japan; kawata@kuhp.kyoto-u.ac.jp (T.K.); ma-imamura@iskweb.co.jp (M.I.); yokuzu@kuhp.kyoto-u.ac.jp (Y.O.); kawait@kuhp.kyoto-u.ac.jp (T.K.); ykuromd@kuhp.kyoto-u.ac.jp (Y.K.); smat522@kuhp.kyoto-u.ac.jp (S.M.); 2 Medical Device Development Division, Ishihara Sangyo Kaisha, LTD, Osaka 550-0002, Japan

**Keywords:** bioactive bone cement, titania, osteoconduction, osteoporosis

## Abstract

Titania bone cement (TBC) reportedly has excellent in vivo bioactivity, yet its osteoconductivity in synovial fluid environments and bone-bonding ability in osteoporosis have not previously been investigated. We aimed to compare the osteoconductivity of two types of cement in a synovial fluid environment and determine their bone-bonding ability in osteoporosis. We implanted TBC and commercial polymethylmethacrylate bone cement (PBC) into rabbit femoral condyles and exposed them to synovial fluid pressure. Rabbits were then euthanized at 6, 12, and 26 weeks, and affinity indices were measured to evaluate osteoconductivity. We generated a rabbit model of osteoporosis through bilateral ovariectomy (OVX) and an 8-week treatment with methylprednisolone sodium succinate (PSL). Pre-hardened TBC and PBC were implanted into the femoral diaphysis of the rabbits in the sham control and OVX + PSL groups. Affinity indices were significantly higher for TBC than for PBC at 12 weeks (40.9 ± 16.8% versus 24.5 ± 9.02%) and 26 weeks (40.2 ± 12.7% versus 21.2 ± 14.2%). The interfacial shear strength was significantly higher for TBC than for PBC at 6 weeks (3.69 ± 1.89 N/mm^2^ versus 1.71 ± 1.23 N/mm^2^) in the OVX + PSL group. These results indicate that TBC is a promising bioactive bone cement for prosthesis fixation in total knee arthroplasty, especially for osteoporosis patients.

## 1. Introduction

Cemented total knee arthroplasty (TKA) is a prevalent surgery for knee deformities, but many issues remain unsolved. A major problem that affects the long-term outcome of this surgery is the subsequent loosening of the prosthesis, which could occur due to a lack of osteoconductivity of the bone cement [1]. Some studies have previously reported that polymethyl methacrylate (PMMA) bone cement, widely used for prosthesis fixation, does not bond directly with the bone. Instead, there is always an intervening fibrous tissue, possibly leading to subsequent aseptic loosening [2,3,4,5].

To solve this issue, various types of bioactive bone cement have been developed [6,7,8,9]. For example, PMMA cement containing bioactive titania fillers (TBC) has been demonstrated to have excellent osteoconductivity and bone-bonding ability in animal studies [10,11,12,13], and TBC has also been demonstrated to have an excellent bone-bonding ability in canine total hip arthroplasty (THA) [14]. It was reported that titania with a specific crystal phase could form hydroxyapatite on the surface [15], and TBC exposed titania fillers on its surface, leading to direct bone-bonding [10].

In cemented TKA, bone cement is clinically exposed to an environment with abundant synovial fluid since the cement–bone interface adjacent to the joint surface is much longer in TKA than in THA. In the knee joint, synovial fluid may invade and disrupt the cement–bone interface, and TBC may not fully demonstrate its bioactivity owing to fluid pressure [16,17]. On the other hand, several studies have suggested a correlation between bone mineral density (BMD) and implant fixation [18,19]; therefore, TBC may not demonstrate optimal bone-bonding ability in osteoporotic bone.

Before using TBC for prosthesis fixation in TKA, it is a prerequisite to demonstrate osteoconductivity or TBC’s bone-bonding ability in a condition that mimics the TKA environment. This study aimed to compare TBC and PBC osteoconductivity in a synovial fluid environment and determine their bone-bonding ability in osteoporotic bone.

## 2. Materials and Methods

### 2.1. Ethics

The Animal Research Committee of Kyoto University approved all experimental animal procedures, and the study conformed with international guidelines on the ethical use of animals (approval number: Med Kyo 14341).

### 2.2. Cement Preparation

The compositions of TBC and PBC are shown in Table 1. TBC contains 20% micron-sized titania particles (Ishihara Sangyo Kaisha, LTD., Osaka, Japan), with a mean size of 3.0 μm and a specific surface area of 2.8 m^2^/g, which are uniformly dispersed in the polymer particles. Details of the polymer particles are as described previously [14].

As an initiator, benzoyl peroxide was added to the powdered component, and as an accelerator, *N,N*-dimethyl-p-toluidine (Tokyo Chemical Industry, Tokyo, Japan) was dissolved in the liquid.

A commercial polymethylmethacrylate bone cement (PBC), Surgical Simplex P Bone Cement (Stryker Howmedica Osteonics, Limerick, Ireland), was used as the control material.

### 2.3. Animal Experiment—Synovial Fluid Environment Model

Twenty-four mature male Japanese white rabbits (weighing 2.5–3.0 kg) were used for the implantation study. They were reared in accordance with the guidelines for the use of experimental animals, as set by Kyoto University, and experiments were performed at the Institute of Laboratory Animals, Faculty of Medicine, Kyoto University. An intravenous injection of pentobarbital (50 mg/kg of body weight) and an intramuscular injection of 60 µg medetomidine were used for anesthesia.

**Figure 1:**
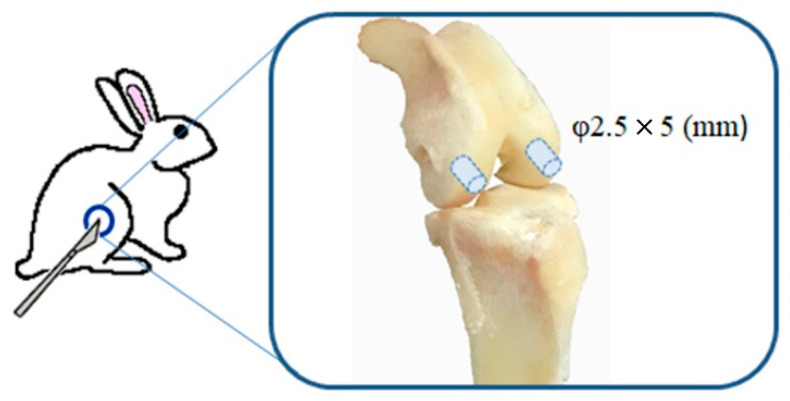
Schematic of the rabbit model used in this study.

**Figure 2:**
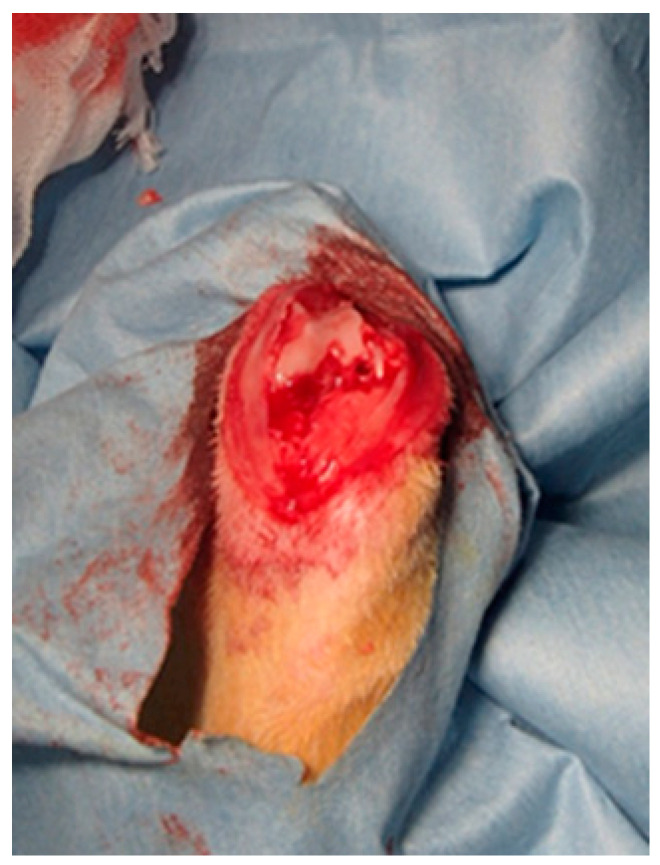
An intraoperative photograph of the rabbit’s femoral condyle. The holes were filled with dough-phase bone cement.

After the rabbits were anesthetized, a midline longitudinal incision was made over the knee joint, and a medial parapatellar approach was applied to the knee. A 2.5-mm stainless steel dental bur was used to create a hole at a depth of approximately 5 mm in the center of both femoral condyles (Figure 1 and Figure 2). After the holes were irrigated with physiological saline and wiped with gauze, they were manually filled with dough-stage bone cement and pressurized manually until the cement polymerized. After lavage, the fascia joint cavity was completely closed before skin closure. Both femurs underwent an operation in the same way, using different cement types in each. Eight rabbits were euthanized postoperatively at 6, 12, and 26 weeks, respectively; there were eight legs containing TBC and PBC, respectively, at each time interval. 

### 2.4. Animal Experiment—Osteoporosis Model

Eight mature female Japanese white rabbits (weighing 2.5–3.0 kg) were used for the osteoporosis study. They were randomly allocated to two groups (a sham control group and an ovariectomy plus methylprednisolone sodium succinate (PSL) injection (OVX + PSL) group). The rabbits were anesthetized in the same way, and OVX + PSL groups received bilateral ovariectomy through a ventral incision. Two weeks after the surgery, rabbits in the OVX + PSL group were injected subcutaneously with PSL at a dosage of 1 mg/kg/day for 8 consecutive weeks [20,21].

After this treatment, the rabbits underwent surgery, and hardened cylindrical cement specimens were inserted under general anesthesia. As reported in a previous study [11], a lateral approach was applied to expose the femoral cortex. Using a dental burr, four holes (2.5 mm in diameter and 12 mm apart) were drilled on the lateral side of each femur through the cortex into the medulla, and cylindrical hardened bone cement was inserted into the holes of each leg with different cement types.

The rabbits were sacrificed 6 weeks after surgery, and a push-out test (described below) was subsequently performed to evaluate BMD and bone-bonding ability.

### 2.5. Micrographic Examination

Sample preparation for micrographic examination was described in detail previously [11]. Briefly, thin vertical cross-sections (100 and 500 µm) were taken from the implant-cortex contact area, and cross-sections (100-µm thickness) were ground to a thickness of about 50 µm for Stevenel’s blue and van Gieson’s picrofuchsin double staining. Cross-sections (500-µm thickness) were polished with a diamond paper, and carbon coating was performed before observation under a scanning electron microscope (SEM) (S-4700, Hitachi, Tokyo, Japan).

For the evaluation of osteoconductivity, we calculated the affinity indices with an optical microscope (DSX500, Olympus Co., Tokyo, Japan). For this study, we used a section of each femur obtained 500 µm from the joint surface. The length of the bone which directly contacted the cement surface, and the total length of the cement surface were measured with DSX-BSW (Olympus Co., ver. 3.1.1.10). The affinity index was calculated as follows:

(1) $$\mathrm{Affinity}\;\mathrm{Index}\;\left(\%\right)=100\;\times \;\frac{direct\;bone\;contact\;length}{total\;length\;of\;the\;cement\;surface}$$

### 2.6. Analysis of Osteoporosis

A cone-beam-type micro-CT system (SMX-100CT-SV-3; Shimadzu Corp., Kyoto, Japan) was utilized to quantify structural parameters of the distal femur. The analytical condition was 55 kV with 50 μA. For all femurs, a 1.5-mm-high region of interest (ROI) was chosen 1.5 mm from the growth plate. After thresholding, BMD was determined at Ratok System Engineering Co., Tokyo, Japan, with no information of the experimental details.

### 2.7. Push-Out Test—Osteoporosis Model

For the osteoporosis model, push-out tests were performed to measure the bone-bonding strength. Four segments, each of which contained a cement specimen, were cut and removed from each femur of the rabbits at 6 weeks after the operation. Then, the specimens were subjected to the push-out test within 2 h of removal. The details of push-out test were described previously [11]. To obtain interfacial shear strength, the maximum load was divided by the contact area between the implant and bone.

### 2.8. Statistical Analysis

Values are expressed in terms of means and standard deviations, and values for each type of cement at each time interval were compared using one-way analysis of variance with Fisher’s least significant difference post hoc test. Statistical assessment was performed using JMP Pro 12 (SAS Institute, Cary, NC, USA). A *p*-value < 0.05 was considered statistically significant.

## 3. Results

### 3.1. Surface Evaluation—Synovial Fluid Environment Model

**Figure 3:**
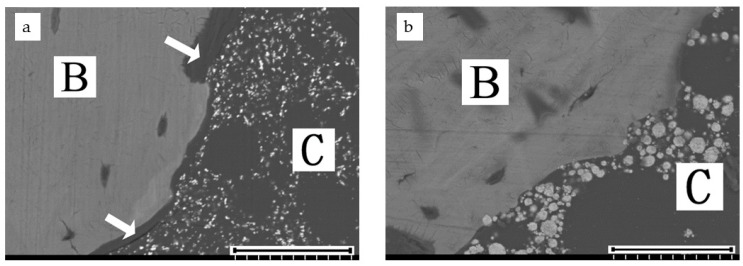
Backscattered SEM images of PBC (**a**) and TBC (**b**) of a rabbit femur at 26 weeks after implantation. B: Bone, C: Cement, Arrows: intervening soft tissue layer. Scale bar: 50 µm.

High-magnification SEM revealed that TBC directly contacted bone at 26 weeks. In contrast, there was an intervening layer of soft tissue (approximately 10 µm in width) in most areas around PBC (Figure 3).

### 3.2. Histological Evaluation—Synovial Fluid Environment Model

**Figure 4:**
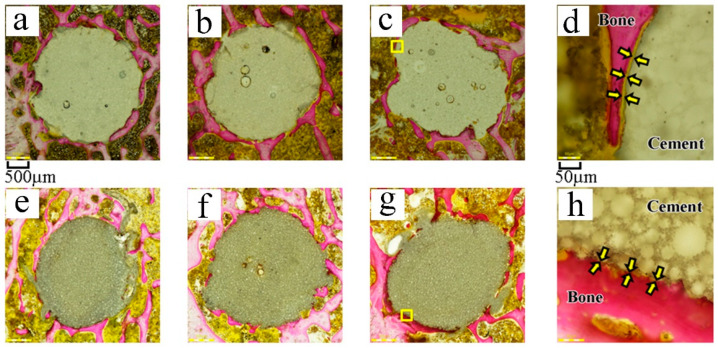
Histological sample of PBC (**a**–**d**) and TBC (**e**–**h**). Samples were stained with Stevenel’s blue and van Gieson’s picrofuchsin double staining. (**a**,**e**): 6 weeks after surgery, (**b**,**f**): 12 weeks after surgery, and (**c**,**g**): 26 weeks after surgery. (**d**,**h**): An enlarged image of the samples shown in (**c**,**g**), yellow squared area. A soft tissue layer (arrows) existed at the bone cement interface in the sample shown in (**d**), but in the sample depicted in (**h**), no such layer was present, and bones were in direct contact with cement. Scale bars: (**a**–**c**,**e**–**g**): 500 µm. (**d**,**h**): 50 µm.

Stevenel’s blue and van Gieson’s picrofuchsin double staining showed bone formation and contact around the cement to be of the same level in rabbits of both TBC and PBC groups at 6 weeks. However, TBC showed more extensive bone contact around the cement at 12 and 26 weeks compared to PBC (Figure 4).

**Figure 5:**
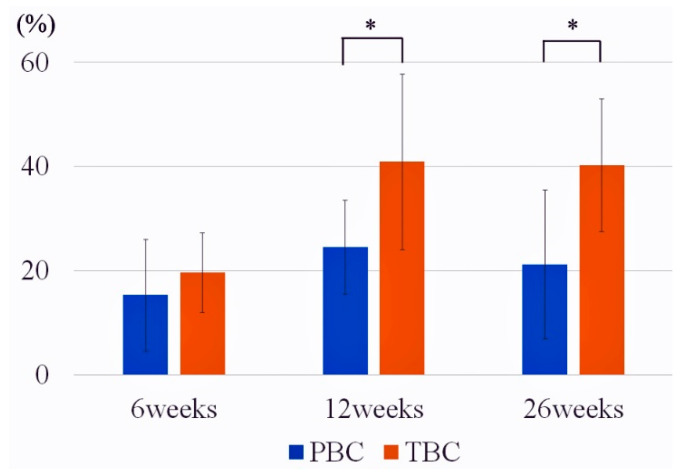
Affinity indices (%) of the PBC and TBC in rabbit femoral condyles at 6, 12, and 26 weeks after implantation (mean ± standard deviation, *n* = 8). * Significant difference (*p* < 0.05).

The mean affinity indices are shown in Figure 5 and Table 2. At 6 weeks, there was no significant difference in the affinity indices between TBC and PBC (*p* = 0.34). At 12 and 26 weeks, the affinity indices of TBC were significantly higher than those of PBC (12 weeks: *p* = 0.0082; 26 weeks, *p* = 0.0024).

Regarding the histological evaluation of the osteoporosis model, sample preparation failed presumably because of excessive dehydration, and good visualization of the cement–bone interface could not be obtained via an optical microscope.

### 3.3. Osteoporosis Evaluation—Osteoporosis Model

No surgical complications, including infection, were observed. BMD in the sham control group and OVX + PSL group was 510.8 ± 11.5 and 458.3 ± 6.76 mg/cm^3^, respectively.

**Figure 6:**
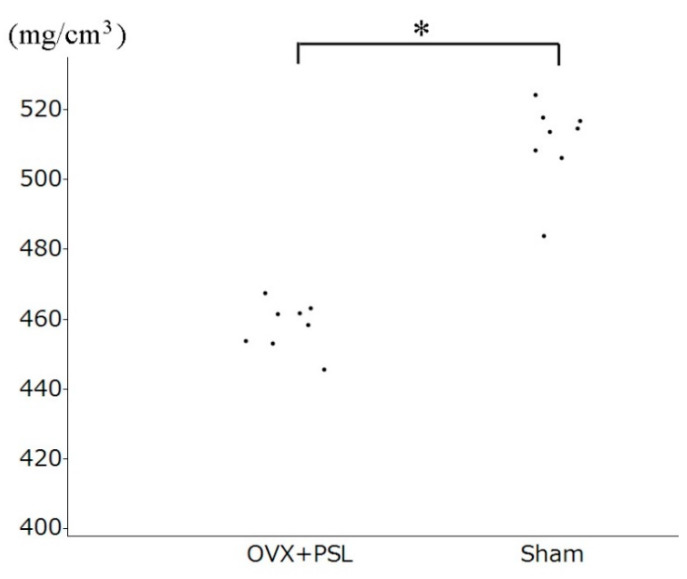
Bone mineral density (mg/cm^3^) in osteoporosis through bilateral ovariectomy (OVX) + PSL and sham control groups 6 weeks after implantation surgery. * Significant difference (*p* < 0.001).

A significant BMD reduction was seen in the OVX + PSL group as compared with the sham control (Figure 6).

### 3.4. Bone-Bonding Evaluation—Osteoporosis Model

**Figure 7:**
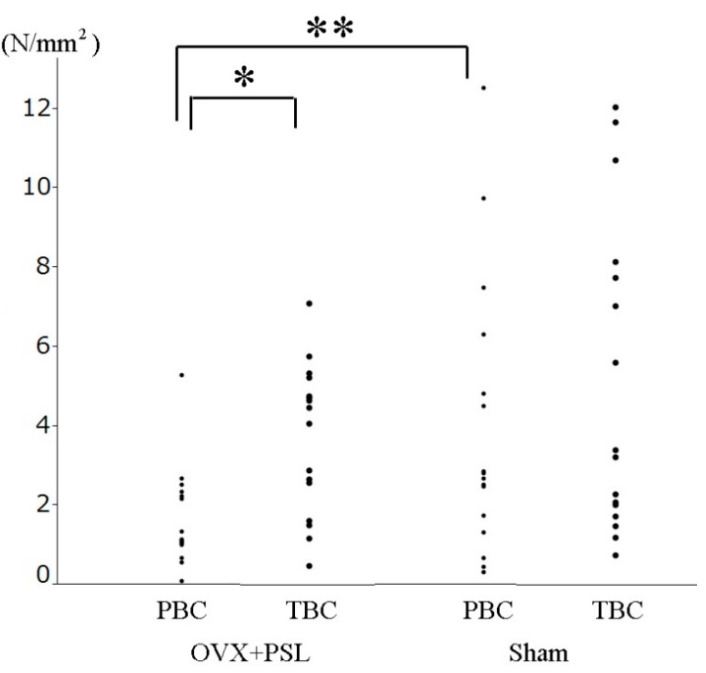
The interfacial shear strength values (N/mm^2^) for PBC and TBC in OVX + PSL, and sham control group. * Significant difference (*p* = 0.019), ** Significant difference (*p* = 0.041).

The interfacial shear strength values for PBC in the sham control and OVX + PSL groups are shown in Figure 7 and Table 3. There was a significant difference between TBC and PBC in the OVX + PSL group and between the sham control group and the OVX + PSL group for PBC. There was, however, no statistically significant difference between the sham control and OVX + PSL groups for TBC (Figure 7).

## 4. Discussion

In this study, we implanted PBC and TBC into the femurs of rabbits and examined the osteoconductivity in a synovial environment and osteoporotic bone. TBC showed significantly better osteoconductivity than PBC at 12 and 26 weeks postoperatively in a synovial environment and significantly higher bone-bonding strength at 6 weeks in osteoporotic rabbit bone. These results indicated that TBC could have good osteoconductivity and bone-bonding ability for therapeutic applications.

The effect of synovial fluid invasion seems to be one of the causes of lower affinity indices in this study. In a previous study, the affinity indices of TBC were 56.5 ± 14.1% at 6 weeks and 67.0 ± 18.1% at 12 weeks [10], and were significantly higher than those in this study. TBC was reported to keep the “dough-phase” long before setting and to have a long working time [11]. TBC and PBC attained a certain penetration of cement into bone, as shown in Figure 4, and the affinity indices of PMMA bone cement in other studies were equivalent to those of PBC in this study [22,23]. Though there were differences in the implantation site and animal species compared to previous studies [10,22,23], the results in this study indicate that synovial fluid invasion has a negative effect on the osteoconductivity of TBC.

In this study, the affinity indices increased between 6 and 12 weeks with both PBC and TBC, but they remained almost the same degree from 12 to 26 weeks. This indicates that bone formation on the cement surface reached an equilibrium around 12 weeks after surgery when the condition of the bone cement interface stabilized and the affinity indices did not change significantly [24].

The backscattered SEM images suggested that exposed titania particles on the surface of TBC acted as a bioactive layer, leading to direct contact between the cement and bone, as reported in previous studies [10,11]. However, there was an intervening layer of soft tissue with 10–30 µm thickness in most areas of the bone cement interface of PBC on the SEM images, even in areas in which the cement surface directly contacted bone tissue at the optical microscope view. TBC had no such marginal layer and showed good osteoconductivity in the knee joint. This result suggested that TBC could have good osteoconductivity when used in TKA.

As for the osteoporosis model, our method of OVX + PSL showed promising results in treating osteoporotic rabbits. Many studies have demonstrated a significant reduction of BMD in rodent models [25]. However, Permay et al. concluded that rabbits are a promising model for osteoporosis research, with several advantages over rodents. The most consistent BMD reduction in the least time was brought about by a combination of ovariectomy and corticoid treatment [20]. This is consistent with our results, showing that the BMD of OVX + PSL group rabbits was less than 470 mg/cm^3^, and all the sham control group rabbits had BMD of more than 500 mg/cm^3^. This suggests that OVX followed by 8 weeks of PSL injection is a reliable method for creating an osteoporotic rabbit model.

In the osteoporosis model, the bone-bonding strength of PBC in the OVX + PSL group was significantly lower than that of the sham control group, while there was no statistically significant difference between the sham control and the OVX + PSL group for TBC. This result indicates that TBC possibly buffered the effect of osteoporosis on bone-bonding strength. However, there were no histological analyses, and the data on bone-bonding strength was only at 6 weeks in this study. Further long-term analyses are necessary to confirm that TBC can hamper osteoporosis’s deleterious effect on bone-bonding strength.

There are several limitations to this study. First, we calculated the affinity indices with optimal microscopic images. As we mentioned above, in some cases, a thin intervening layer of soft tissue lay at the cement–bone interface on the SEM images, even in areas where the optical microscopic images showed the cement surface to be in direct contact with the bone tissue. In this study, we did not use high-magnification SEM images for evaluating affinity indices because the low titania content made it time-consuming and difficult for us to estimate the affinity index on backscattered SEM images. The use of high-magnification SEM images might have allowed us to assess TBC and PBC osteoconductivity precisely. Second, we could not perform histological analyses in the osteoporosis model for technical mistakes in sample preparation and only investigated the bone-bonding strength at 6 weeks due to financial limitations. The BMD measurements were costly, and we could not further increase the sample size. It should be highlighted that only four rabbits for each group were examined without histology at 6 weeks in the osteoporosis model, and obtained data are insufficient to demonstrate TBC efficacy in osteoporosis. Further histological studies are required to investigate the characteristics of the interface between TBC and bone in osteoporosis.

## 5. Conclusions

TBC was demonstrated to have higher osteoconductivity than PBC in rabbit femora connected to the synovial fluid environment and possibly higher or comparable bone-bonding ability in osteoporotic bone. Therefore, TBC is a promising bioactive bone cement for prosthesis fixation in TKA, especially for osteoporosis patients.

## Figures and Tables

**Table 1:** Composition of TBC and PBC.

Powder	TBC	PBC
Titania	20 *w*/*w*	-
Barium sulfate	-	6.7 *w*/*w*
Polystyrene/MMA copolymer	42.5 *w*/*w*	50 *w*/*w*
PMMA	7.5 *w*/*w*	10 *w*/*w*
Benzoyl peroxide	5.4 wt% of MMA.	(a) *
**Liquid**	-	-
MMA	30 *w*/*w*	32.5 *w*/*w*
*N,N*-dimethyl-p-toluidine	0.95 wt% of MMA.	0.9 *w*/*w*
Hydroquinone	70 ppm	1.5 mg

(a) * Benzoyl peroxide was included in polymer. TBC, titania bone cement; PBC, commercial polymethylmethacrylate bone cement. MMA, methyl methacrylate.

**Table 2:** Affinity index of each group (*n* = 8).

Cement	6 Weeks	12 Weeks *	26 Weeks *
TBC	19.6 ± 7.68	40.9 ± 16.8	40.2 ± 12.7
PBC	15.3 ± 10.7	24.5 ± 9.02	21.2 ± 14.2

Values are reported as the mean ± standard deviation (%). * Significant difference between groups (12 weeks: *p* = 0.0082; 26 weeks, *p* = 0.0024).

**Table 3:** The interfacial shear strength values in the sham control and OVX+PSL groups. (*n* = 16).

Cement	Sham	OVX + PSL *
TBC	5.08 ± 3.95	3.69 ± 1.89
PBC **	3.94 ± 3.49	1.71 ± 1.23

Values are reported as the mean ± standard deviation (N/mm^2^). * Significant difference between TBC and PBC (*p* = 0.019), ** Significant difference between Sham control and OVX + PLS group (*p* = 0.041).

## Data Availability

The data presented in this study are available on request from the corresponding author. The data are not publicly available due to the privacy policy of Ishihara Sangyo Kaisha, LTD.
